# The efficacy of TACE combined sorafenib in advanced stages hepatocellullar carcinoma

**DOI:** 10.1186/1471-2407-12-263

**Published:** 2012-06-21

**Authors:** Xu-Dong Qu, Cheng-Shi Chen, Jian-Hua Wang, Zhi-ping Yan, Jie-min Chen, Gao-quan Gong, Qin-xin Liu, Jian-jun Luo, Lin-xiao Liu, Rong Liu, Sheng Qian

**Affiliations:** 1 Department of Radiology, Zhong Shan Hospital, Fudan University, Fenglin Road, Shanghai, 200032, People’s Republic of China

## Abstract

**Background:**

The long-term survival in hepatocellullar carcinoma (HCC) patients after transarterial chemoembolization (TACE) remains dismal due to local and/or regional recurrence as well as distant metastasis. The efficacy of sorafenib in advanced HCC has been demonstrated and brought great hope. Recently, the use of sorafenib in combination with TACE for BCLC stage B and C HCC patients was recommended. However, data on this dual-modality treatment is little, and its advantage over TACE alone has not been addressed. The present study sought to understand the efficacy of the combination of TACE and sorafenib in the treatment of advanced HCC.

**Methods:**

Between June 2008 and Feb 2011, 45 patients with advanced HCC were enrolled and treated with sorafenib in combination with TACE according to an institutional protocol of the Zhongshan hospital, Fudan University. The control group of 45 other HCC patients with similar characteristics treated with TACE alone in the same period of time in our institute were selected for retrospective comparison of the treatment outcomes especially overall survival time. Adverse reactions induced by sorafenib were observed and recorded.

**Results:**

The median overall survival time of the combined treatment group was 27 (95% Confidence Interval: 21.9–32.1) months, and that of TACE alone group was 17 months (95% Confidence Interval: 8.9–25.0) months (*P* = 0.001). Patients required significantly less frequent TACE for their symptomatic treatment after the initiation of sorafenib therapy. The most common adverse events associated with sorafenib were hand-foot skin reaction, rash and diarrhea. Of CTCAE grade IV or V toxicity was observed.

**Conclusion:**

TACE combined sorafenib significantly prolonged median overall survival time of patients with advanced HCC.

## Background

Hepatocellular carcinoma (HCC) is one of the most common and fatal gastrointestinal malignancies. The incidence of HCC in China is particularly high, accounting for 55% of all cases diagnosed worldwide [1,2]. The definitive treatment modality for HCC is surgical resection. However, complete resection is not feasible for the majority of patients because of advance disease when diagnosed [3-5]. Transarterial chemoembolization (TACE) is generally accepted as an effectively palliative treatment for patients with unresectable HCC^.^ Despite of the efficacy in local disease control and symptomatic relief, long-term survival in HCC patients after TACE remains dismal due to local and/or regional recurrence, as well as distant metastasis [6-10]. Clearly, effectively systemic treatment for advanced HCC is urgently needed.

Sorafenib is a multikinase inhibitor with effects on tumor proliferation and angiogenesis [11]. The efficacy of sorafenib in advanced HCC has been demonstrated repeatedly in phase II and phase III randomized trials [11,12]. In a recently published phase II clinical trial, the use of sorafenib in combination with TACE for BCLC stage B and C HCC patients was recommended. However, data on this dual-modality treatment is little, and its advantage over TACE alone has not been addressed. The aim of this report is to bolster the existing but limited data by documenting the outcome of patients with advanced HCC treated with TACE and sorafenib. The treatment outcomes of this group of patients were also compared in a retrospective fashion with another group of HCC patients with similar characteristics in a case controlled setting.

## Methods

Between June 2008 and Feb 2011, 45 patients with advanced stages of HCC (BCLC-B, C) were enrolled and treated with sorafenib in combinatin with TACE(TACE + sorafenib group) according to an institutional treatment protocol at the Zhongshan hospital, Fudan University. All patients were diagnosed by histology, cytology, or persistently elevated serum alpha-fetoprotein (AFP ≥ 400 ng/ml) with typical imaging findings. Histological or cytological confirmation for the diagnosis of HCC was mandatory for patients with AFP < 400 ng/ml. Other inclusion criteria for this protocol included (1) unresectable multinodular asymptomatic HCC unsuitable for surgical resection according to the Barcelona Clinical Liver Cancer (BCLC) staging classification, and (2) Child-Pugh class A and B without encephalopathy with ECOG Performance Status of 0-1. An additional group of 45 HCC patients with similar characteristics including age, gender, BCLC stage of the disease, Child-Pugh classification, and ECOG performance status were selected and matched at 1:1 ratio for a retrospective comparison of the treatment outcome.

### Treatment

#### Transarterial chemoembolization

Angiography of celiac, hepatic, superior mesenteric, left gastric, and inferior phrenic arteries was performed to identify all feeding arteries of the tumor. A 2.7–5.0 F catheter was then inserted into the target artery. Oxaliplatin (100–200 mg) and/or fluorouracil glycosides (500–1000 mg) were infused followed by epirubicin (30–60 mg) mixed with 5–25 ml of iodized oil under fluoroscopic monitoring. The mixture was infused at a rate of 0.5–1 ml/min until stasis flow in tumor vascularity was achieved. Finally, gelatin sponge or 300–500um micosphere was used to embolize the feeding artery of tumor.

#### Sorafenib treatment

Patients who were treated with sorafenib were prescribed with two tablets of sorafenib (200 mg tablet) twice daily. The recommended dose adjustment that reduced the dose to the lowest level according to CTCAE would be used. When the drug-related adverse events panished, whether taking sorafenib 400 mg twice daily were decided according to the different types of the adverse events by clinical doctors.

### Follow-up

All patients treated in our center for HCC were required to be followed up according to our institutional protocol. Each follow-up session includes a detailed history and physical examination, ECOG performance status classification, Child pugh score evaluation, and an abdominal enhanced CT/MRI scan. All patients were followed up at a 6- to 8-week interval.

### Statistical analysis

The primary objective of the current study was overall survival (OS), which refers to the time between first TACE to death by any cause. The treatment outcomes of the HCC TACE group were compared with the TACE combined with sorafenib group. Survival analysis was estimated by the Kaplan-Meier survival method and compared by the log-rank test. All statistical tests were two-sided, P < 0.05 was considered statistically significant. Statistical analysis was performed with Statistical Product and Service Solutions computer software for Windows (SPSS Inc, Chicago, IL).

## Results

### Characteristics of patients and disease

The characteristics of patients and their diseases including age, gender, stage, Child-Pugh classification, AFP level prior to treatment, tumor type and size, prior history of hepatitis, liver function, as well as the presence of PVT and/or metastasis are listed in Table 1. There is no significant difference in any of the characteristics between the two groups of patients.

**Table 1:** The basic demographic and disease characteristics

	**TACE + Sorafenib**		**TACE**	**P**
	**(*n* = 45)**		**(*n* = 45)**	
Age	51 ± 11.7		49 ± 11.0	>0.05
Men/women	41/4		41/4	>0.05
PVT	20		23	>0.05
Distant metastasis	11		12	>0.05
Prior surgery	19		14	>0.05
ECOG				>0.05
	0	43	41	
	1	2	4	
Child pugh				>0.05
	A	33	35	
	B	12	10	
	C	0	0	
AFP				>0.05
	<400 ng/ml	16	19	
	≥400 ng/ml	29	26	
Diagnosis of Hepatitis				>0.05
	HBV	38	37	
	HCV	0	0	
	None	7	8	
Tumor size, n (%)				
	>5 cm			
		35(77.78)	36(80.00)	
	≤5 cm			
		10(22.22)	9(20.00)	
Tumor type, n (%)				>0.05
	Massive			
		13(28.89)	10(22.22)	
	Multifocal			
		16(35.56)	18(40.00)	
	Diffuse			
		16(35.56)	17(37.78)	
BCLC stage				>0.05
	A	0	0	
	B	16	17	
	C	29	28	

TACE: transcatheter arterial chemoembolization; TACE-S: TACE combined with sorafenib; BCLC: Barcelona Clinic Liver Cancer; ECOG PS: Eastern Cooperative Oncology Group Performance Status; HBV, hepatitis B virus; HCV, hepatitis C virus; AFP, alpha-fetoprotein, AST, aspartate aminotransferase.

### Survival

**Figure 1:**
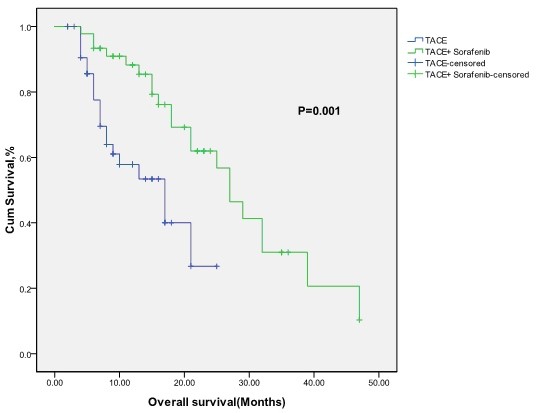
Kaplan-Meier overall survival functions for TACE + sorafenib group and TACE group.

The median survival time of the combined treatment group was 27 (95% Confidence Interval 21.9–32.1) months and that of TACE alone group was 17 (95% Confidence Interval 8.9–25.0) months (*P* = 0.001) (Figure 1).

**Figure 2:**
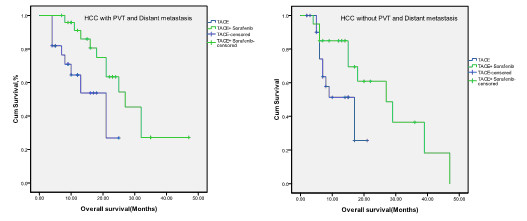
Kaplan-Meier overall survival functions for TACE + sorafenib group and TACE group in patients with PVT and distant metastasis and without PVT and distant metastasis separately.

The median survival time of patients with portal vein thrombosis and/or distant metastasis was shorter than those who had neither (Table 2). The addition of sorafenib significantly improved the overall survival of both group of patients. Furthermore, when sorafenib was added to TACE, the significance of portal vein thromobosis/metastasis on overall survival diminished (27.2 vs. 29.1 months, *P* = 0.558) (Figure 2).

**Table 2:** Comparison of the median survival time of patients with or without portal vein thrombosis and/or distant metastasis

	**Groups**	***n***	**Mean (95% Confidence Interval)**	***P*value**
HCC without portal vein thrombosis/metastasis	TACE	22	15.94 (11.87–20.01)	0.027
	TACE + Sorafenib	25	29.09 (22.76–35.41)	
HCC with portal vein thrombosis/metastasis	TACE	23	12.00 (9.96–16.02)	0.041
	TACE + Sorafenib	20	27.20 (19.07–35.32)	

### Adverse effects (adverse events related to sorafenib)

No grade IV or V toxicity occurred in either group of patients. Table 3 details the incidence of all grade 1–3 (CTCAE v. 3.0) toxicities secondary to sorafenib observed based on the Common Terminology Criteria for Adverse Events (CTCAE) version 3.0. Commonly observed adverse effects in our group of patients received sorafenib included fatigue, skin reaction, hear loss, nausea/anorexia, diarrhea, hypertension, depression, and muscle ache.

**Table 3:** The adverse events after intaking of sorafenib [the number of cases (%)]

**CTCAE grade**	**I**		**II**		**III**	
**Symptom**	**Number (%)**		**Number (%)**		**Number (%)**	
Hand-foot skin reaction	10	(22.2)	15	(33.3)	12	(26.7)
Rash	19	(42.2)	5	(11.1)	2	(4.44)
Diarrhea	17	(37.8)	4	(8.89)	1	(2.22)
Hair loss	17	(37.8)	2	(4.44)	2	(4.44)
Hypertension	15	(33.3)	7	(15.6)	3	(6.67)
Fatigue	22	(48.9)	3	(6.67)	0	(0)
Anorexia	12	(26.7)	2	(4.44)	0	(0)
Nausea	11	(24.4)	1	(2.22)	0	(0)
Depression	9	(20)	0	(0)	0	(0)
Muscle aches	14	(31.1)	0	(0)	0	(0)

Adverse-effects induced by TACE included nausea/vomiting, tenderness in the right upper abdomen, fever, and transient LFT dysfunction. However, no grade 4 or above TACE-induced adverse-effect was observed.

TACE was administrated in each group of patients, and in the group of combination therapy sorafenib was initiated after at least one TACE procedure. The duration time of taking sorafenib is 11.61 ± 5.3 months. All patients in both treatment groups experienced all grades of adverse events induced by TACE, of which some patients experienced grade 3 adverse events based on CTCAE 3 and return to CTCAE grade 1 after proper treatment. Grade 4 or above adverse events was not observed in both groups. In consideration of the possible overlaying of adverse events induced by TACE and sorafenib, sorafenib was interrupted for 3 days prior to TACE and at least 3 days after TACE during the combination therapy, and return to dosing when liver functions, the blood test and embolism syndrome CTCAE assessment reached CTCAE grade 1 or lower.

## Discussion

Patients’ outcomes with TACE have been recently improved based on the application of micro-catheter technique and doxorubicin-eluting beads technique. The survival benefits of TACE were better in patients with focal liver lesions, hypervascular tumors and without vascular invasion [4]. TACE was usually not performed in the case of multiple lesions, hypovascular tumor, and vascular invasion even extra-hepatic disease. The limitation of TACE was the incomplete target lesion necrosis, which made patients require repeated TACE treatments. In addition, residue tumor proliferation, tumor recurrence and metastasis after TACE influenced long-term outcome [13,14]. Comprehensive therapy based on combination with systemic therapy played an important role in improving the efficacy of therapy for advanced HCC. The development of molecular targeted agent sorafenib, one of systemic therapies, brought hope for HCC patients.

Sorafenib, an orally active multikinase inhibitor with effects on tumor-cell proliferation and tumor angiogenesis, was initially identified as a Raf kinase inhibitor, by inhibiting the serine-threonine kinase Raf-1 and B-Raf. It also inhibits vascular endothelial growth factor receptors (VEGFR) 1, 2, and 3; platelet-derived growth factor receptor β(PDGFR); and RET receptor tyrosine kinases. Sorafenib inhibits MEK and ERK phosphorylation, down-regulates cyeline D1 level, reduces eIF4E phosphorylation and down-regulates anti-apoptosis protein Mc11 [15,16]. In SHARP and Oriental trials, monotherapy with sorafenib significantly prolonged overall survivals (44% and 47% respectively) and delayed time to progression (73% and nearly 1 fold respectively) in patients with advanced HCC compared with that in placebo recipients. Moreover, treatment with sorafenib was well tolerated and safe. Based on these data, sorafenib was recommended as the standard treatment for advanced HCC. However, the two trials also showed that the efficacy of monotherapy with sorafenib was limited since the absolute benefit in survival time compared with placebo was not so prominent.

In view of liver primary lesion, portal vein invasion and distant metastasis, there has been a consensus on comprehensive therapy based on combination therapy for intermediate-advanced HCC. The efficacy of the combining use of sorafenib and TACE in patients with advanced HCC including those with BCLC stage C disease was suggested in a recently published phase II clinical trial [17]. Close to 60% of patients achieved objective response and the treatment was well tolerated although 40 sorafenib dose interruptions were observed. However, patients’ survival has not been reported.

The enrolled patients in our study were those of unresectable HCC, BCLC stage B or C. Baseline characteristics were well balanced between the study groups. The median survival time in the group treated with sorafenib plus TACE was 27 (95% Confidence Interval: 21.89–32.10) months, while the median survival time in the group of TACE alone was 17 (95% Confidence Interval: 12.66–29.33) months (Figure 1). Our data indicated that sorafenib could prolong the median survival time of patients with HCC treated with TACE.

We also observed that there was no significant difference in the median survival time between patients with portal vein thrombosis and distant metastasis and those without portal vein thrombosis and distant metastasis in combination therapy group. Although there was probably certain statistics deviation due to the small sample size, it is still suggested that sorafenib could cover the shortage of TACE to improve the outcome of patients with vascular invasion and distant metastasis.

Various adverse events occurred during sorafenib treatment in clinical practice, and significant individual differences were also found. However, no grade 4 adverse events were observed and the most common grade 3 events were hand-foot skin reactions and hypertension. Patients received full guidance during the treatment, thus adverse events were relieved after additional prevention, treatment and dose adjustment of sorafenib. Discontinuation due to adverse events was not observed. Collectively, the combination of sorafenib and TACE do not increase sorafenib related adverse events.

Although patients received sorafenib + TACE were accrued and treated according to our institutional protocol, the comparison between those with or without sorafenib therapy were retrospective in nature. Therefore, during the selection of patients treated with TACE alone, efforts were applied to avoid selection bias and the characteristics of patients and their diseases matched well in our two groups. Furthermore, as the survival data of patients treated with sorafenib in combination with TACE were not available at the time of our study design, sample size calculation for a phase II clinical trial was not possible. We consider retrospective nature of the comparison as well as a small sample size of 45 patients two substantial limitations of the current study.

The results of our current study and the above-mentioned phase II trial provided the only documentation of the efficacy and safety of the combination of sorafenib and local treatment using TACE. Clearly, these results are far from conclusive. The survival advantage of the combination of sorafenib and TACE over sorafenib alone has never been addressed. Furthermore, the optimal use of this combination, especially the optimal timing of TACE during sorafenib use needs to be studied. A number of recently published trials indicated that the use of external beam radiotherapy or I-125 implant might further improve local control of the intrahepatic disease [18,19]. Whether the combination of sorafenib with TACE and/or radiotherapy can further improve patients’ survival also needs further investigation.

## Conclusion

The combination of sorafenib and TACE produced a median survival time of 27 months in patients with advanced HCC, significantly longer than 17 months of patients treated with TACE alone. Sorafenib also reduced the frequency of TACE required for intrahepatic symptomatic control. The combination of sorafenib and TACE was well tolerated with no severe adverse events observed in our group of patients. Further studies preferably in a randomized fashion are required to compare the efficacy of the combination of sorafenib and TACE to either sorafenib or TACE, as well as the observation of the optimal sequence of such combination.

## Competing interests

The authors declare that they have no competing interests.

## Authors’ contributions

XD Q and CS C contributed to acquisition of data, analysis and interpretation of data, performing the statistical analysis; JH W contributed to conception and design, and drafting the manuscript; ZP Y helped to draft the manuscript; JM C, GQ G, QX L, JJ L, LX L, R L; S Q contribute to Collection of cases. All authors read and approved the final manuscript.

## Pre-publication history

The pre-publication history for this paper can be accessed here:

http://www.biomedcentral.com/1471-2407/12/263/prepub
